# Spatial temperature gradients guide axonal outgrowth

**DOI:** 10.1038/srep29876

**Published:** 2016-07-27

**Authors:** Bryan Black, Vivek Vishwakarma, Kamal Dhakal, Samik Bhattarai, Prabhakar Pradhan, Ankur Jain, Young-tae Kim, Samarendra Mohanty

**Affiliations:** 1 Biophysics and Physiology Lab, Department of Physics, USA; 2 Department of Mechanical & Aerospace Engineering, USA; 3 Department of Bioengineering, The University of Texas at Arlington, TX 76019, USA; 4 Department of Physics, University of Memphis, TN 38152, USA; 5 Department of Urology, UT Southwestern Medical Center, TX, USA; 6 Nanoscope Technologies, TX 76012, USA

## Abstract

Formation of neural networks during development and regeneration after injury depends on accuracy of axonal pathfinding, which is primarily believed to be influenced by chemical cues. Recently, there is growing evidence that physical cues can play crucial role in axonal guidance. However, detailed mechanism involved in such guidance cues is lacking. By using weakly-focused near-infrared continuous wave (CW) laser microbeam in the path of an advancing axon, we discovered that the beam acts as a repulsive guidance cue. Here, we report that this highly-effective at-a-distance guidance is the result of a temperature field produced by the near-infrared laser light absorption. Since light absorption by extracellular medium increases when the laser wavelength was red shifted, the threshold laser power for reliable guidance was significantly lower in the near-infrared as compared to the visible spectrum. The spatial temperature gradient caused by the near-infrared laser beam at-a-distance was found to activate temperature-sensitive membrane receptors, resulting in an influx of calcium. The repulsive guidance effect was significantly reduced when extracellular calcium was depleted or in the presence of TRPV1-antagonist. Further, direct heating using micro-heater confirmed that the axonal guidance is caused by shallow temperature-gradient, eliminating the role of any non-photothermal effects.

Functional neural circuitry formation requires highly accurate axonal pathfinding during neural development or regeneration. The direction of axonal outgrowth is dictated by the detection and integration of competing guidance cues found in the surrounding environment. These guidance cues can be chemical^1^ or physical. Physical cues are in general electrical^2^ and optical^3^) in nature or any combination thereof. Focused laser beam has enabled modulation and generation of several chemical^4^,^5^ and physical (force^3^,^6^ and fluid flow^7^,^8^,^9^) cues to influence axonal guidance. In general, the light-based methods for axonal guidance can be divided into two categories, indirect and direct. In the indirect category, light is used as an indirect guidance cue, where focused laser beams have been used to induce photolysis, asymmetrically releasing calcium^4^ near the growth cone, or in which circularly polarized light has been used to trap and rotate birefringent particles, steering nearby growth cones by microfluidic flow^7^,^8^ or use in photolabile hydrogel^10^. The direct category includes purely-optical attractive guidance cue exploiting the optical forces due to a focused laser beam, impinging upon the leading edge of the neuronal growth cone^6^. There have been several variants of the attractive optical guidance cue, which include various beam profiles^3^,^6^, wavelengths, tapered optical fibers^11^, and temporal patterns^12^. Recently, we found purely-optical repulsive-guidance of primary axons by near infrared (NIR) light to be highly effective^13^,^14^. The ability to guide axons using purely-optical attractive^3^,^6^,^11^ or repulsive^13^,^14^ means is very promising due to light’s potential for high spatial^15^ and temporal selectivity, its absolute sterility, and for its minimal invasiveness.

Until recently, the focus of a majority of axonal guidance studies have been towards the understanding of neuronal systems’ response to different chemical cues^1^. However, there is a lack of detailed investigation and evidence of mechanism involved in guidance of axons with physical cues. This may be due to the fact that until recently^13^,^14^ effective and long-range guidance of primary axons by physical cues was not demonstrated. For example, although direct optical attractive guidance methods have been demonstrated for a decade, the proposed mechanisms by which light can directly influence the steering of axonal growth cones have never been fully understood or developed. The widely-varying proposed mechanisms include optical forces acting on intracellular components such as globular actin monomers, optical forces orienting and stabilizing existing filopodia, and temperature rise. For the most part, increase of temperature has been disregarded as a probable primary mechanism for attractive axonal guidance since the temperature increases due to absorption by culture medium, cellular membrane, and intracellular component would be relatively small when using NIR light. Nevertheless, as we have recently reported^13^,^14^, a focused CW NIR laser spot, asymmetrically positioned in front of advancing primary rat cortical axons (or goldfish retinal ganglion cell axons), has effectively served as a purely-optical *repulsive* guidance cue. Since the laser spot is not directly impinging upon the growth cone, we have concluded that a laser-induced diffusive field is responsible for this repulsive guidance effect. The guidance cues are primarily detected by the axon’s splayed distal end, called the growth cone, where the membrane expresses a wide variety of dense, specialized receptors. When a guidance cue gradient is applied across the growth cone, membrane receptors closer to the source of the gradient will have a higher probability of activation. This leads to an asymmetric rate of intracellular signaling cascades, which are largely mediated by secondary messengers. Over the time, the growth cone becomes polarized toward or away from the source of the gradient, depending on whether the guidance cue is attractive or repulsive.

One important secondary messenger is calcium (Ca^2+^). Intracellular Ca^2+^ levels have long been known to play an important role in axonal growth and guidance^4^,^16^, as well as being implicated in central nervous system neuronal migration^17^,^18^. Intracellular Ca^2+^ flux can be the result of internal Ca^2+^ stores being released from the endoplasmic reticulum, or the result of extracellular Ca^2+^ influx^19^,^20^, triggered by the opening of cation-permeable membrane channels, such as transient receptor potential (TRP) channels^20^,^21^. Only a narrow range of global intracellular Ca^2+^ concentration is optimal for growth cone extension. Higher or lower intracellular concentrations slow down the growth cone extension, or even cause the retraction and the growth cone collapse^16^,^22^,^23^. Therefore, whether a guidance cue is attractive or repulsive is largely dependent on the local intracellular Ca^2+^ gradient which the guidance cue evokes. Under normal conditions, a steep intracellular Ca^2+^ gradient resulting from a guidance cue (e.g. temperature, as we hypothesized) gradient is likely to result in attraction, whereas a shallow intracellular calcium gradient is likely to result in repulsion^4^,^24^. Blocking Ca^2+^ influx by inhibiting membrane cation channels or changing the extracellular calcium concentration baseline has been shown to abolish the guidance response, or even switch a normally attractive guidance cue to a repulsive one^24^.

In recent years, the transient receptor potential (TRP) family has been frequently implicated in axonal guidance events, either as the primary mechanism, or as a mediator for downstream molecular cues^19^,^25^. Transient receptor potential vanilloid (TRPV) channels are a sub-family of TRP proteins, which form polymodal, non-selective cation channels on the membranes of specific cells. These channels are known to open in response to capsaicin, low pH, protons, various lipids, and, in some cases (TRPV1–4), heat^26^,^27^. High expression levels of TRPV channels are found in sensory neurons, where they are primarily responsible for mammalian temperature sensation^26^,^28^,^29^. However, TRPV1–4 are also expressed in the central nervous system, with TRPV1–3 being expressed in the cortical regions of mammalian brains^30^. TRPV1, arguably the most extensively studied among the TRPV subfamily, is often cited as having an activation temperature of 40–43 °C^26^,^27^,^31^,^32^,^33^. However, several research groups have shown TRPV1 activation to occur at lower, more physiologically relevant temperatures (35–39 °C)^25^,^34^, depending on environment and the strength of applied temperature gradient.

Here, we quantitatively validate our hypothesis that very weak temperature gradients are capable of significant repulsive guidance of cortical axons. The involvement of temperature in axonal guidance is confirmed by (i) the lower threshold laser power required for guidance while using NIR wavelength(s) as compared to visible light, (ii) a significantly reduced response to the thermal cue (generated by light at-a-distance) in the presence of a TRPV1 antagonist, and (iii) repulsive guidance of axons by direct heating (using micro-heater), on the scale of 0.1 °C across the growth cone.

## Results

**Figure 1:**
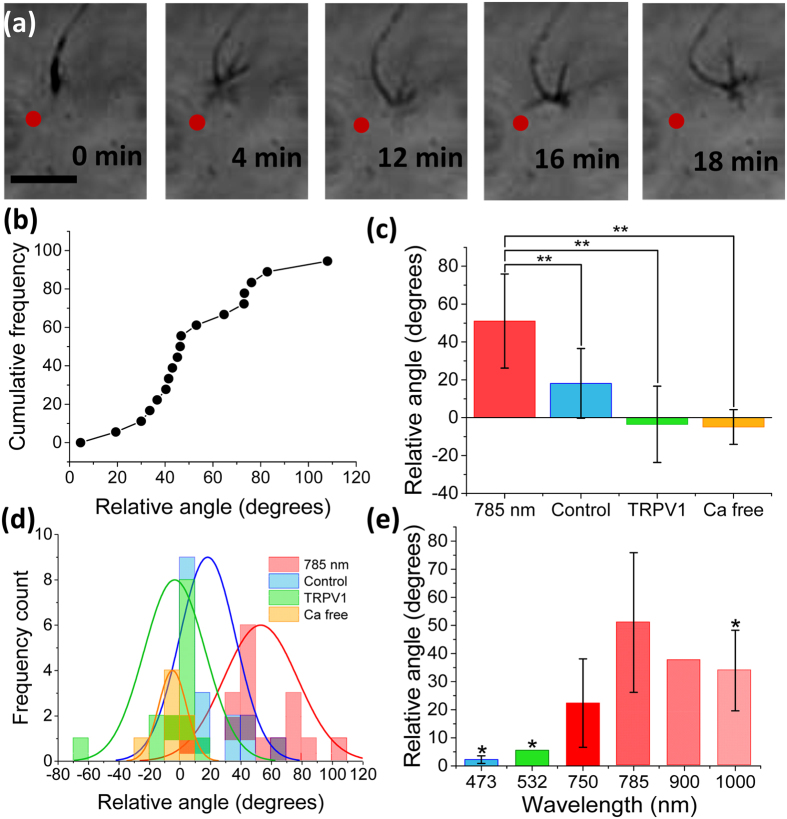
Mechanism of light-induced repulsive axonal guidance. (**a**) Time-lapse images of primary cortical axon responding to asymmetrical positioning of weakly-focused (0.5 NA) laser (80 mW at 785 nm) spot (marked by red spot). Scale bar: 10 μm. (**b**) Cumulative frequency (in %) of relative turning angle in response to single guidance by a static laser spot. (**c**) Relative angle turned in the cases of positive control (785 nm, laser on, red), negative control (laser off, blue), silenced (785 nm, laser on, 10 μM SB-366791, TRPV1 antagonist, green), and calcium-free (785 nm, laser on, Ca-free medium, orange) guidance trials. Statistical significance between trial groups (**p < 0.01). Average ± S.D. (**d**) Frequency counts for the four major experimental groups. Lines indicate a 100% scaled normal fit. (**e**) Relative angle turned in response to the laser cue at various wavelengths (473, 532, 750, 785, 900, 1000 nm). *Sample-site laser power at 473, 532, or 1000 nm were <80 mW (used for 750, 785, 900 nm). Angles reported are from trials in which the axon grew to the static position of the laser spot.

In order to examine the at-a-distance guidance effect of a weakly-focused CW near infrared (NIR) laser beam on primary rat cortical neurons, the laser spot (sample site power: 60–80 mW at 785 nm) was asymmetrically positioned ~5 μm from the leading edge of advancing growth cones (n = 27). Of the 27 positive control experiments performed, 18 growth cones ultimately extended beyond the static position of the laser spot, and were considered in further analysis. 89% of these advancing growth cones were effectively guided away from the guidance cue source (laser spot) by a significant angle (Fig. 1a), as compared to the mean of negative control (no laser applied). Figure 1b shows the cumulative frequency for positive control experiments. These findings based on weakly-focused (spot size > wavelength) NIR laser beam at-a-distance are consistent with those previously reported in the case of tightly-focused (100x) NIR laser-based repulsive guidance^13^,^14^. The mean turning angle for positive control experiments (785 nm, 60–80 mW) was 51 ± 24.9 degrees, whereas the mean turning angle for negative control experiments (no laser) was 18.1 ± 18.4 degrees (Fig. 1c).

Next, we tested whether repulsive laser-based guidance effects could be eliminated by blocking temperature sensitive TRPV1 calcium channels, or by reducing the extracellular calcium concentration. Silencing experiments were conducted in the presence of 10 μM SB-366791, an established TRPV1 antagonist^30^,^35^,^36^,^37^. These experiments resulted in a dramatic decrease in the axonal guidance response to the laser guidance cue (n = 17). Of the 17 trials, 14 axons were observed to grow beyond the static position of the laser spot with an average turning angle of −3.5 ± 20.2 degrees (Fig. 1c). Many of the TRPV1 treated axons interacted directly with the laser spot, even growing directly through the laser spot in several instances. Several filopodia appeared to become optically-trapped following laser interaction, similar to the results of attractive laser-based guidance experiments^3^. In order to evaluate the role of calcium, the guidance experiments were performed after replacing the cell culture medium with calcium-free medium. The repulsive guidance effect was significantly reduced while using calcium-free medium (Fig. 1c), resulting in an average turning angle of −4.9 ± 9.2 degrees (n = 7). This conclusively implies that the guidance mechanism is not only mediated by calcium influx, but that TRPV1 channels are intimately involved in the process. Time-lapse images of examples from each trial group are shown in Suppl. Fig. 1. The statistical significance of the positive control was tested against the negative control, TRPV1-silencing, and calcium-depletion experimental data groups. A one-way ANOVA F-test, performed in Origin, identified the population means as significantly different (Prob > F = 9.4 × 10^−10^). Individual two-tailed t-tests were then performed between the positive control and other groups with Welch correction, identifying the positive control as statistically significant (e.g. P = 9.7 × 10^−5^ when compared with negative control). In Fig. 1d, we show the frequency counts for the four major experimental groups.

**Figure 2:**
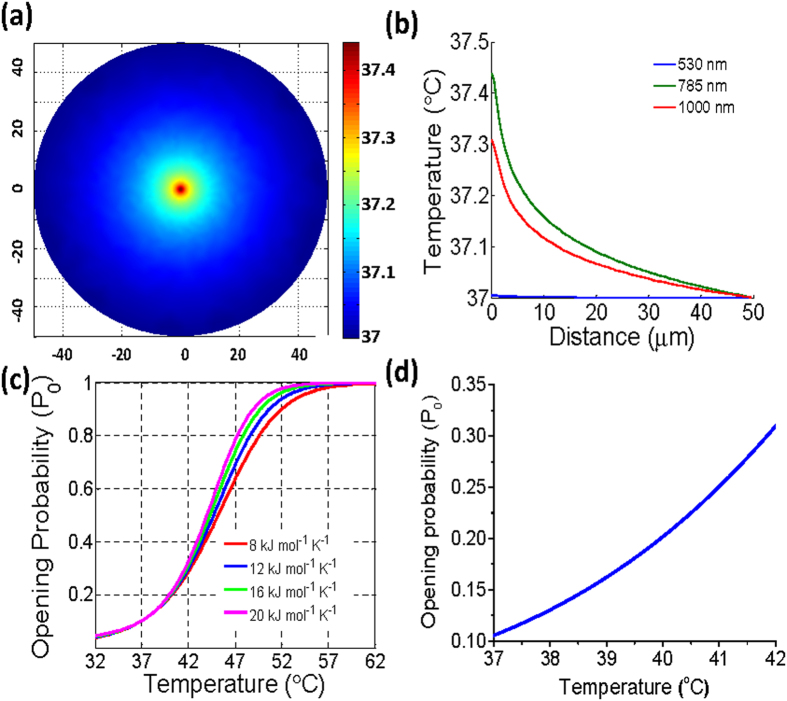
Simulation of temperature increment and induced TRPV1-channel opening-probability. (**a**) Temperature field plot (X, Y: in μm) for laser beam (785 nm, 80 mW) weakly-focused (0.5 NA) in water-medium. Color bar is in °C. (**b**) Radial line plot of temperature field due to weakly-focused (0.5 NA) laser spot at 785 nm (80 mW, green), 1000 nm (10 mW, red), and 530 nm (10 mW, blue). (**c**) Temperature-dependent opening-probability of TRPV1-channel for specific heat variances (ΔC_p_) of 8 (red), 12 (blue), 16 (green), and 20 (turquoise) kJ mol^−1^ K^−1^. (**d**) Zoomed region of temperature-dependent opening-probability of TRVP1 for our experimentally-relevant temperature range.

To further confirm the role of photothermal effects on the observed repulsive axonal guidance, laser-based guidance experiments were performed at various wavelengths, spanning 473 to 1000 nm. In this spectral range, the temperature rise due to absorption of the laser beam by extra-cellular medium is known to vary by more than an order of magnitude. The relative angle turned at the NIR wavelengths (750–1000 nm) were significantly higher (p < 0.01) than that due to visible (473, 532 nm) laser beams (Fig. 1e). Further, the relative turning angle while using 10 mW of 1000 nm laser beam was not statistically significant to that achieved using 80 mW of 750–900 nm laser beams. These two findings suggest that temperature rise caused by laser beam (larger in case of 1000 nm due to absorption of water-medium) is a key parameter for the observed repulsive axonal guidance. Figure 2a shows the simulation-based temperature field plot for weakly-focused (0.5 NA) laser beam (785 nm, 80 mW) in water-medium. Our infrared camera measurements show a temperature rise of approximately 0.4 °C (Suppl. Fig. 2) within 100 seconds after laser was on in a selected region of interest where the laser beam was weakly-focused. Thus, the simulations and measurements confirm the magnitude of the temperature rise due to the absorption of the NIR laser beam by the medium. Though we could not deliver positive control level (60–80 mW) sample-site laser power at all reported NIR wavelengths, it is important to note that our simulations show that 10 mW laser power at 1000 nm generates a similar temperature rise to that using 80 mW at 785 nm (Fig. 2b). However, 10 mW of visible (530 nm) laser beam (in water) could not generate detectable increase in temperature (Fig. 2b). In the case of 785 and 1000 nm light, the theoretical temperature difference across the width of a neuronal growth cone (20 μm), located 5 μm from the center of the laser spot is approximately 0.25 °C. Since TRPV1 channel was implicated (Fig. 1) in the photothermal based repulsive guidance, we investigated the temperature-dependent opening-probability of the TRPV1-channels. Figure 2c shows the results of the simulations on probability of opening of TRPV1 channel for different specific heat variances (ΔC_p_). The zoomed region of temperature-dependent opening-probability of TRVP1 for our experimentally-relevant temperature range is shown in Fig. 2d. According to our temperature-dependent TRPV1 opening probability simulations, the minute temperature difference (0.25 °C) caused by the NIR laser beam corresponds to an increase of the opening probability of ~10% across the growth cone. This increase in TRPV1 opening-probability, integrated over the observed turning period (15–30 min), may be sufficient to allow significant calcium influx resulting in the repulsive laser-based guidance.

**Figure 3:**
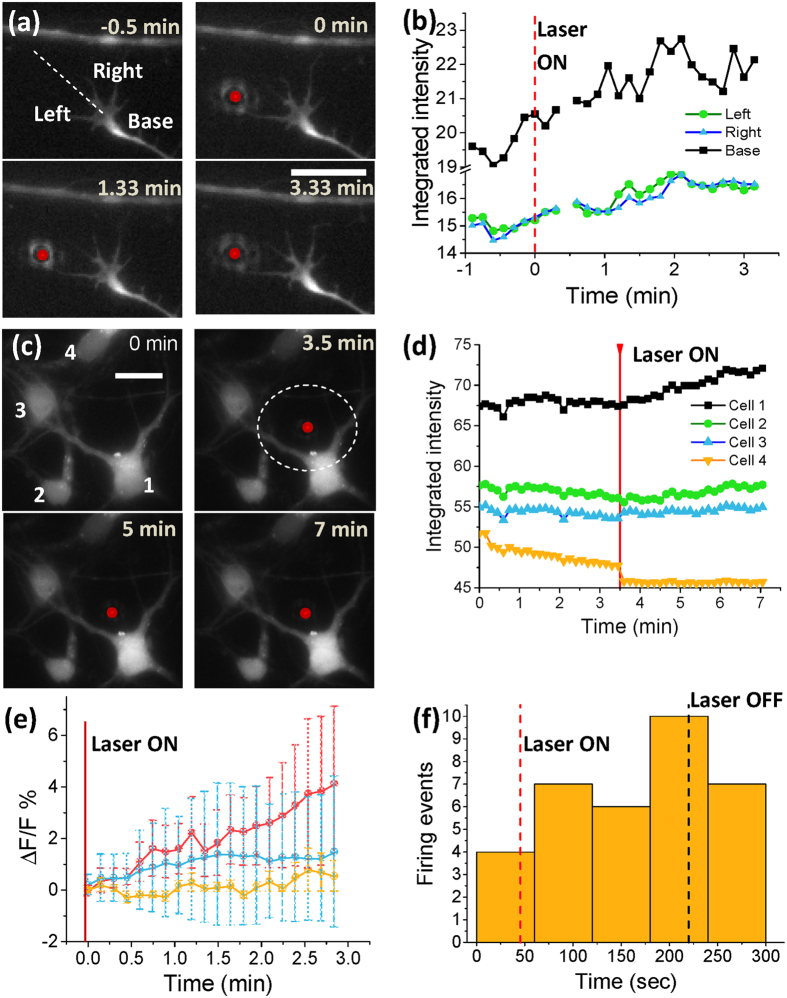
Calcium imaging of growth cone and soma of rat cortical neurons in response to NIR laser spot at-a-distance. (**a**) Representative epifluorescence time-lapse images of cortical neuron growth cone incubated with Fluo-3 AM (5 μM) and exposed to 1064 nm (10 mW) focused (1.3 NA) laser spot at-a-distance (marked by red dot). Left, right, and base of the growth cone shown in first panel. Scale bar: 10 μm. (**b**) Dynamics of fluorescence intensity integrated over three regions of interest (left, right, and central base) within the growth cone in response to laser spot at-a-distance. (**c**) Time-lapse fluorescence images of four cortical neurons (denoted by 1–4) incubated with Fluo-3 AM (5 μM) and exposed to 1064 nm (10 mW) focused (1.3 NA) laser spot at-a-distance (marked by red spot). (**d**) Dynamics of fluorescence intensity integrated over ROIs of equal area in the soma of the four cortical neurons before and after exposure to laser spot. Laser-on time indicated with red line. (**e**) Kinetics of fractional percentage change in integrated fluorescence intensity (ΔF/F %) for ROIs in cortical neurons in response to laser spot (1064 nm, 10 mW, 1.3 NA) for three different neuronal regions from the laser spot (distance: 0–10 μm, red; 10–20 μm, blue; >20 μm, orange). The dotted circle in panel c represents region within distance of 0–10 μm from the laser spot. N = 11. Average ± S.D. (**f**) Change in firing rate (measured by calcium fluorescence spikes) in response to laser spot at-a-distance (10 mW, 1000 nm).

In order to determine whether the observed repulsive guidance effect is mediated by an influx of intracellular calcium, we conducted laser guidance trials in which neurons were incubated for 10–15 min with 5 μM of Fluo-3 AM, a well-established fluorescent intracellular bound-calcium indicator^17^,^22^,^38^. Fluorescence experiments were first conducted on cortical growth cones. Figure 3a shows a time-lapse series in which a clear increase in the fluorescence of the growth cone can be observed following application of laser guidance cue (1064 nm, 10 mW, 1.3 NA) at-a-distance. Dynamics of calcium fluorescence intensity, integrated over three regions of interest (left, right, and central base) within the growth cone, in response to laser spot at-a-distance is shown in Fig. 3b. Since the expression of temperature-sensitive ion channels is not restricted to the growth cone, we decided to also perform fluorescence calcium influx imaging of the soma in response to laser spot at-a-distance. Figure 3c shows a clear increase in fluorescence for the neuron-1, nearest to the laser spot. Figure 3d summarizes the dynamical change in the fluorescence intensity for four cortical neurons within the field of view before and after switching on the NIR laser beam at-a-distance. The fluorescence increase was observed to be not significant (none in some cases) as the distance of the neuron from the laser spot increased. Figure 3e shows the kinetics of fractional percentage change in integrated fluorescence intensity (ΔF/F %) for three different neuronal regions from the laser spot (distance: 0–10 μm, 10–20 μm, >20 μm). There is a clear increase in ΔF/F for neurons in the nearest regions, whereas ΔF/F in the farthest regions is closer to their respective baselines (i.e. no laser) (Fig. 3e). Laser-induced temperature field simulations (Fig. 2a,b) illustrate that the temperature is expected to sharply decay to the baseline value within 20 μm from the center of the laser spot. The significant calcium fluorescence increase following application of the NIR laser beam at-a-distance supports our temperature-based, TRPV1-mediated, calcium-rise led guidance hypothesis. Furthermore, the firing rate of spontaneously-spiking neuron was found to be modulated in response to NIR laser spot at-a-distance (Suppl. Fig. 3). Following 45 seconds of baseline (no laser), when the laser beam was applied near the soma (Suppl. Fig. 3a) the calcium spiking rate increased (Fig. 3f). Following 180 seconds of observed increase in spiking activities, the laser was turned off and the spiking rate was found to be reduced, mirroring previous studies which reported increased firing rates with increasing temperature^26^.

**Figure 4:**
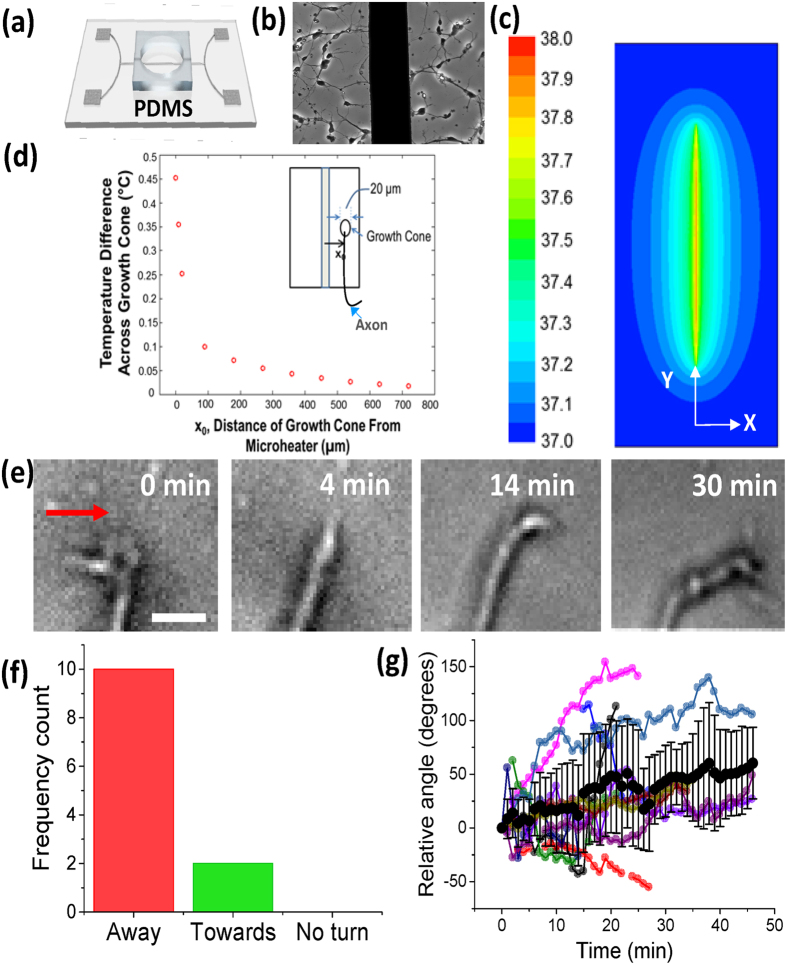
Direct heating induced shallow temperature gradient leads to axonal repulsion. (**a**) Illustration of titanium micro-heating device and PDMS confinement well (not to scale). (**b**) Phase contrast image of cultured primary cortical neurons near titanium heating element (60 μm width, central vertical black strip). (**c**) Simulation of temperature field plot (in XY) after passing 1 mA current through a titanium heater (60 μm width) submerged in medium (water). (**d**) Line profile (along X) of temperature-difference across growth cone (width assumed to be 20 μm) versus distance from the titanium micro-heating element. Inset: Axon growing parallel to heating element, left side of the growth cone is at distance (X_0_–10) μm and right side is at (X_0_+10) μm. (**e**) Representative time-lapse phase contrast (20x) images of axonal repulsion in response to temperature rise of 1 °C at heater-medium interface (out of the field of view at a distance of 200 μm to the left). The red arrow indicates direction of high to low temperature. (**f**) Frequency count for advancing axons turning away from the micro-heater, towards the micro-heater, or which exhibited no turn (<10 degrees). (**g**) Kinetics of turning angle for advancing axons during direct heating experiments. N = 12, Average ± S.D. (black circles and error bars). Individual axons represented by other colors.

Finally, in order to rule out all other non-thermal effects due to light’s interaction with the neuron or substrate, direct-heating devices were fabricated to evaluate the effect of shallow temperature-gradient on cortical neuron guidance. A 1 mA current was passed through a 60 μm wide titanium heater line microfabricated on a glass slide (Fig. 4a, and Suppl. Fig. 4a) containing cortical neurons in a PDMS well (Fig. 4b). The calibrated electrical resistance of the titanium micro-heater as a function of ambient temperature provides the temperature rise from the measured electrical resistance during guidance experiments (Suppl. Fig. 4b). Further, the temperature field due to direct heating by passing electric current through the micro-heater is calculated to reach the steady state condition within ~100 ms (Suppl. Fig. 4c). As a result, the growth cones are exposed to the steady-state temperature distribution for the entire duration of the experiment. The peak steady-state temperature in the micro-heater device could be controlled by a change in the electric current passing through the titanium micro-heating element (Suppl. Fig. 4d). The current of 1mA corresponds to a 1 °C temperature rise (37 to 38 °C) at the micro-heater-medium interface which decays sharply to the baseline temperature (~37.05 °C) within 1 mm (Fig. 4c). Figure 4d shows the simulated line profile of the temperature difference across growth cone (assumed to be 20 μm in width) as a function of distance away from the micro-heater for 1mA current in water. As shown, the temperature gradient across the growth cone was found to decrease sharply as the distance from the micro-heater increases. For growth cones located close to the micro-heater, the temperature gradient can be as large as 0.4 °C. Beyond 100 μm from the micro-heater, the temperature gradient across the growth cone is 0.1 °C or less.

To obtain the effect of a weak temperature gradient (≤5 °C/mm) on axonal guidance, direct heating based guidance of axons growing within 100–400 μm in either direction from the titanium micro-heater was measured (n = 12). As shown in Fig. 4d, in this region the temperature difference across growth cone is ≤0.1 °C. Figure 4e shows a representative time-lapse series of images demonstrating the repulsive guidance induced by the shallow temperature rise (initiated at t = 0 min). 83% of advancing axons ultimately turned away from the titanium micro-heater within 1 hr (Fig. 4f). Figure 4g shows the kinetics of turning angle for advancing axons during direct heating experiments. A steady increase in the average relative turning angle with time observed, with the average repulsive guidance angle reaching 45.3 ± 33.3 degrees.

## Discussion

Both spectrally-tuned laser- and microheater-based guidance experimental results showed significant axon repulsion. Furthermore, silencing TRPV1 channels and depleting the extracellular calcium independently abolished the repulsive axonal guidance response. We have successfully demonstrated that weak temperature gradients, on the scale of 0.1 °C across the width of the growth cone (≤5 °C/mm), can cause repulsive guidance in primary mammalian cortical axons.Although the TRPV1 activation threshold temperature is commonly reported to be between 40–43 °C^26^,^27^,^28^,^31^,^32^,^39^, several researchers have demonstrated that inward currents due to TRVP1 activation begin at lower, more physiologically relevant temperatures^25^,^34^. In a report by Sharif-Naeini *et al*.^36^ showed that when ~2 min temperature ramps from 36 to 38 °C were applied to vasopressin neurons via heated solution perfusion, inward current responses and significant firing rate differences in these neurons were observed as compared to PVZ neurons (which do not express TRPV1 channels) and TRPV1-silenced experiments (1 μM SB-366791). Furthermore, since TRPV1 and TRPV3 channels are known to co-express and heteromultimerize^21^ in the mammalian cerebral cortex, silencing of TRPV1 channels may non-selectively modulate the TRPV3 channels (which are activated at 35–39 °C)^40^, or activate TRPV1/TRPV3 hybrid channels. Additionally, it has been shown that when thermal fluctuations near the membrane are significant, the resulting thermal motions can drive or reduce the force of actin polymerization, affecting lamellipodia protrusion/extension^41^.

It should be noted that our findings are not contradictory to those reported results on attractive, laser-based optical guidance studies^3^,^6^,^11^. We believe that they are, in fact, complementary. Apart from optical forces stabilizing actin filaments (and/or microtubules) and/or aligning the filopodia (in case of laser spot overlapping the growth cone), the strength of the intracellular calcium gradient is known to be largely responsible for the determination of whether a guidance cue is attractive or repulsive. Experiments^3^,^6^,^11^ in which the laser is focused directly on the growth cone are reasonably expected to induce steeper intracellular calcium gradients. Decreased in the separation between the sample and laser spot will only increase the temperature and temperature gradient across the growth cone, increasing the opening probability of temperature-sensitive membrane channels. Additionally, optical forces due to direct impingement of the laser spot on the growth cone may be activating stretch-sensitive calcium ion channels^42^. The resulting calcium influx sum may bring local intracellular calcium concentrations into the optimal “attractive” range. Figure S5 illustrates our proposed (photo)thermal mechanisms involved in attractive and repulsive axonal guidance depending on the distance of the growth cone from the laser spot. Dependence of laser-induced axonal guidance parameters (direct forcing on actin/filopodia, activity of stretch-activated calcium ion channels, and temperature gradient) on distance of growth cone from the laser spot is shown in Fig. S5c. Based on the amount of calcium induced by these guidance parameters, we can define the attractive and repulsive regimes as a function of the distance of growth cone from the laser spot.

The observed shallow temperature-gradient based guidance responses are on the scale of temperature gradients of those measured during embryonic development. Therefore, it might be plausible that such shallow temperature-gradients affect neurogenesis or early neural development. During development, blood vessels and neurons share same outgrowth cues and physically grow together. Therefore, due to blood flow weak temperature gradient is expected to exist which can influence the axonal guidance. Thermal guidance might also lead to new therapeutic strategies for guided nerve regeneration or selective re-innervation. Dynamically-controllable spatially-distributed complex temperature gradients can now be realized following recent microfabrication strategies^43^,^44^.

## Conclusions

To conclude, we have discovered that central nervous system axons can sense and can be guided by shallow temperature-gradient, an important physical cue. The involvement of temperature in axonal guidance is confirmed by tuning the laser wavelength and use of temperature-sensitive ion-channel antagonist, as well as direct heating on the scale of 0.1 °C across the growth cone. This has significant physiological relevance in addition to other physical cues namely force^3^ and fluid flow^7^,^8^ which were earlier demonstrated to be involved in axonal guidance. Physical cues such as temperature, pressure, flow patterns, and shear stress are known to exist during embryogenesis and act on the vascular network in a number of ways, and may contribute to the development of neuronal network. The mechanotransduction by these physical cues may also trigger the promotion or repression of certain genes, which are responsible for axonal outgrowth.

## Methods

The experimental protocol involving animals was approved by The University of Texas at Arlington’s Institutional Animal Care and Use Committee. The methods were carried out in accordance with the relevant guidelines.

### Cell extraction and culture

All experimental procedures were conducted according to Institutional Animal Care and Use Committee approved protocol. Primary cortical neurons were isolated from embryonic 18-day (E18) rat embryos. The cortical tissues were dissected, cleaned of the meningeal layer, and enzymatically dissociated (0.125% trypsin in L-15 medium) for 20 minutes at 37 °C. The dissociated rat-derived cortical neurons were seeded (100,000/device) on Poly-D-lysine (PDL, 0.01%, Sigma) pre-coated glass coverslips with an affixed Polydimethylsiloxane (PDMS, Sylgard 184, Dow corning) barrier well. Neurons were cultured in serum-free medium (Neurobasal medium supplemented with B-27, BDNF, and NT-3, 10 ng/ml), which was replaced every 3 days for the duration of the sample’s viability.

### Laser-based axonal guidance

A near-infrared laser beam, emitting from a tunable Ti: Sapphire laser operating in CW mode, was expanded to a diameter of 7 mm and guided via folding mirrors through a 1:1 telescope, an electro-mechanically controlled shutter, and a rotatable polarizer towards the rear port of an inverted microscope (Nikon Ti: Eclipse). The laser beam was reflected by a dichroic mirror towards the back aperture of the microscope objective, and the light from a halogen lamp was transmitted through an emission filter in order to remove the back-reflected laser beam reaching the CCD camera. In our set-up, while 80 mW sample-site power could be achieved at 750–900 nm, only 10 mW sample-site power is achievable at 1000 nm and visible wavelengths. Repulsive optical-based guidance experiments were conducted using both 100x (Nikon, 1.3 NA, oil immersion) and 20x (Nikon, Ph1, 0.5 NA) microscope objectives. The microscope’s mechanical stage, filter wheels, and condenser were enclosed by a temperature-controlled incubator which was maintained at 37 °C throughout the duration of experiment. Cortical neurons, cultured on coverslip, were enclosed in stage-top incubators which contained water wells (to maintain humidity), and input/output pipes for flow-through of premixed 5% CO_2_.

TRPV1 silencing experiments were conducted with positive control parameters, in the presence of 10 μM SB-366791, a well-established TRPV1 antagonist. Cells were allowed to incubate with the antagonist for 1 hour at 37 °C prior to the experiments. Positive control experimental conditions were also reproduced in the presence of calcium-free cell culture medium. Normal Neurobasal medium was removed from the PDMS wells and cortical neurons were washed once with the calcium-free medium. PDMS wells were then filled with calcium-free medium for the duration of the guidance experiments.

All images were collected by an EMCCD (Cascade, Photometrics) and analyzed in ImageJ software (NIH). Relative guidance angles were measured from a common reference angle with ImageJ’s angle tool. Deviations from the reference angle toward the center of the guidance cue were considered negative, whereas deviations away from the center of the guidance cue were considered positive. Since no laser cue was present during negative control experiments, turns toward the left of the field of view were taken to be negative, whereas turns toward the right were taken to be positive.

### Experimental measurement of temperature rise by MWIR camera

Experimental measurement of the temperature increment due to 785 nm laser absorption was carried out using a mid-wave infrared (MWIR) thermal camera (FLIR SC6000), which detects light in the wavelength range of 1.0–5.0 μm. Light counts are collected and converted to temperature readings with an accuracy of ±2%, based on factory calibration. Empty cell culture devices, identical to those used during guidance experiments, were filled with culture medium and exposed to positive control laser parameters (785 nm, 80 mW) following 20 seconds of baseline. Negative control parameters (no laser) were also carried out using the MWIR camera. Measurements reflect the temperature rise within a region of interest (circle of radius 200 μm, centered on laser spot).

### Simulations of laser-induced temperature fields

Finite element simulations were carried out to determine the magnitude and distribution of the temperature field due to a focused laser spot. The temporal- and spatial-dependent temperature rise is given by the standard heat diffusion equation:


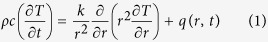


where the source term *q*(*r, t*) is proportional to the z-dependent intensity (I_o_(r, t)) of a focused, Gaussian laser beam multiplied by the linear absorption of the cell culture medium (water), *α.*


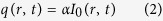


*I*_0_ is related to the measured laser power at the sample site (*P*) and the radial distance from the laser spot center:


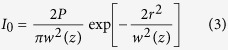


where *w*(*z*) is the *z*-dependent beam waist radius, with *z* measured from the laser focal plane. In this study, we have reduced the problem to two-dimensions, assuming the laser spot is focused at the interface between the glass and cell culture medium domains, and neglecting medium absorption at higher *z* values. In that case


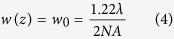


The resulting second order differential equation was solved in Matlab using the inbuilt PDE toolbox. Dirichlet boundary conditions were imposed at the geometrical boundary limits of the cell culture medium domain (circular, 1 mm diameter). The triangular mesh was initialized and refined by the PDE toolbox to have mesh growth of 1.3 from the center of the source term.

For these simulations, we have assumed that all the energy absorbed by the medium goes towards heating and we neglected the higher order absorption of culture medium proteins, pH indicators (phenol red), and the direct absorption of scattered light by cells or cellular components. The absorption of 785 nm light by the glass coverslip is assumed to be negligible compared to that of the cell culture medium (*α* < 0.01).

### Simulating temperature-dependent TRPV1 opening probability

The probability that a protein changes its conformational state is relatable to the Gibb’s free energy difference between the two states (open and closed), and is therefore necessarily a function of temperature.


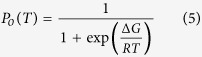


The temperature dependence of the Gibb’s free energy difference can be expressed by the well-known thermodynamic equality


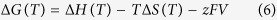


where *ΔH* is the temperature dependent enthalpy change, *ΔS* is the temperature dependent entropy change, *z* is the gating charge, *F* is Faraday’s constant, and *V* is the transmembrane potential. The expressions for enthalpy and entropy are also temperature dependent functions, which are related to the specific heat capacity changes (*ΔC*_*p*_) with temperature while at constant pressure.


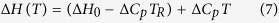



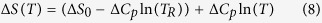


where *ΔH*_0_ and *ΔS*_0_ are the enthalpy and entropy values at the reference temperature (*T*_*R*_). These values, along with *ΔC*_*p*_ are experimentally determined values, and different parameter values have been taken from previous experimental findings^45^. Since there is some discrepancy in experimental determinations of *ΔC*_*p*_ in the physiological range of temperatures, we have plotted the temperature dependent opening probability for *ΔC*_*p*_ spanning the range of reported values (8–20 kJ mol^−1^ K^−1^). Simulations were carried out in Matlab 209A using standard interpolation for Equation (5).

### Calcium fluorescence imaging

1 mM stock Fluo-3 AM was mixed with warm (37 °C) Neurobasal medium to a 5 μM final concentration. The cell culture medium was then replaced with Fluo-3/Neurobasal solution and allowed to incubate for 15 minutes at 37 °C. Cells were washed for five minutes with fresh, warm Neurobasal medium. After washing, Neurobasal medium was replaced by warm low-fluorescent Hibernate E medium (BrainBits) for imaging. A mercury lamp served as the excitation source, emitted light of which passed through an electro-mechanically controlled filter wheel (on/off) and then via a blue excitation filter before being coupled to the back aperture of the microscope objective (100x, 1.3 NA). Green fluorescence images (through a band pass emission filter) were collected by the EMCCD and processed/analyzed with ImageJ software. Stacked time-lapse image sequences were analyzed by the ImageJ ROI Manager, which measured the selected region’s integrated fluorescence intensity. All regions of interest used in the analysis of a single experimental image sequences were of equal size.

### Micro-heater fabrication

Micro-heater devices comprising a thin metal heater on standard glass slides were fabricated using photolithography and metal deposition in a class 100 clean room facility. Titanium was used as the micro-heater material because of its biocompatibility, as well as high electrical resistivity, which provides the capability of large heat generation. In addition, titanium also has a large temperature coefficient of resistivity, which makes it an ideal material for simultaneous use as a temperature sensor. Glass slides were ultra-cleaned prior to microfabrication using a piranha solution (3H_2_SO_4_:H_2_O_2_), followed by cleaning steps in acetone, methanol and DI water. Photolithography was then carried out in an OAI manual front/backside contact mask aligner. Deposition of 0.2 μm titanium was carried out in an AJA ATC ORION evaporator. Finally, lift-off in acetone resulted in the definition of metal features on the substrate. The fabricated micro-heating element is 1.8 cm long and 60 μm wide.

### Micro-heater calibration

In addition to its use as a heating device, the titanium micro-heater can also be used for temperature measurement based on resistance thermometry. Calibration of the micro-heater was carried out prior to its use in experiments, by measuring the electrical resistance of the titanium micro-heater as a function of temperature. To do so, the microfabricated device was mounted on an Instec HCS622V thermal stage capable of precise temperature control. The resistance of the micro-heater line was measured as a function of temperature, by passing a small electric current from a Keithley 2612A source meter, and measuring voltage across the micro-heater line using a Keithley 2100 voltmeter. The test current was chosen to be small enough not to cause significant self-heating, and yet provide measurable voltage. As shown in Fig. S4b, heater resistance was measured to be a linear function of temperature. Figure S4b shows that a measurement of resistance during a heating experiment can be used to precisely determine the temperature of the micro-heater.

### Simulations of micro-heater induced temperature fields

Finite element simulations were carried out to determine the nature and magnitude of temperature gradient around the micro-heater line. The temperature distribution in the micro-heater device is governed by the energy conservation equation given by


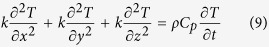


where *T* is the temperature increase relative to the ambient temperature.

Equation (9) was solved using a finite-element simulation code with a mesh containing at least 300,000 nodes, in order to ensure grid independence of results. Joule heating due to electric current passing through the micro-heater line was modeled, and a natural convection was assumed on the top of the micro-heater device. The entire device was assumed to be at 37 °C prior to application of the heating current.

Figure S4c plots the temperature as a function of distance away from the micro-heater line for six different times, indicating the evolution of the temperature distribution with time, starting from the application of the electric current. This figure shows that thermal steady state is reached within around 100 ms. Figure S4d plots the steady-state temperature as a function of distance away from the heater, indicating a sharp temperature gradient in the vicinity of the heater. This temperature profile decays away to the ambient temperature as distance from the heater increases. Further, Fig. S4d shows that the peak temperature in the micro-heater device can be controlled by changing the magnitude of the electric current.

### Micro-heater based experimental design for axonal guidance

Based on our NIR laser guidance experiments, temperature rise of <1 °C was required to investigate the behavior of cortical neurons in a temperature field. The appropriate electric current required for a specific temperature rise was determined by measuring the micro-heater resistance upon passage of the electric current, and using the calibration plot in Fig. S4b to determine the micro-heater temperature. Based on the accuracy of calibration, and the least count of the sourcemeter, the accuracy of temperature measurement of the micro-heater line during experiment was around 0.1 °C. A Keithley 2612A source meter was used during experiments to source the heating current through the micro-heater, while a Keithley 2100 voltmeter was used to monitor the electrical resistance, and hence the temperature of the micro-heater line.

## Additional Information

**How to cite this article**: Black, B. *et al*. Spatial temperature gradients guide axonal outgrowth. *Sci. Rep.* **6**, 29876; doi: 10.1038/srep29876 (2016).

## Supplementary Material

Supplementary Information
