# Supplementary material for: Spatial temperature gradients guide axonal outgrowth
Source: Sci Rep. 2016 Jul 27;6:29876. doi: 10.1038/srep29876 (PMC4962095; doi:10.1038/srep29876)
Supplement: Supplementary Information [file srep29876-s1.pdf]

Supplementary Information

Spatial temperature gradients guide axonal outgrowth

Bryan Black[1], Vivek Vishwakarma[2], Kamal Dhakal[1], Samik Bhattarai[3], Prabhakar Pradhan[4], Ankur Jain[2], Young-tae Kim[3], Samarendra Mohanty[1,5*]

[1]Biophysics and Physiology Lab, Department of Physics;

[2]Department of Mechanical & Aerospace Engineering; [3]Department of Bioengineering, The University of Texas at Arlington, TX 76019.

[4]Department of Physics, University of Memphis, TN 38152.

[5]Nanoscope Technologies, TX 76012.

*To whom correspondence should be addressed:

Dr. Samarendra Mohanty,

Nanoscope Technologies, LLC,

2519 Radcliffe Dr., Arlington, TX 76012, USA.

Email: smohanty@nanoscopetech.com

Tel.: 949-439-7923

Fax: 817-719-2692

Supplementary Figure captions

Suppl. Fig. 1. Time-lapse images of representative axonal guidance trials for positive control (785 nm), negative control (Control, no laser spot), TRPV1 blocker (10 µM SB-366791, TRPV1 antagonist), and calcium-free medium (Ca^{2+} free). Laser focus spot positions are indicated with red circle. The solid white line represents the direction of axonal outgrowth in that frame. The initial direction of axonal outgrowth is shown by dashed white line. Scale bar: 15 µm.

Suppl. Fig. 2. Temperature as a function of time for positive (Black line, 785 nm, 80 mW, 0.5 NA, in water medium) and negative (Red line, no laser) controls, as measured by MWIR camera. Measurements reflect temperature increase within a region of interest (200 µm radius circle) centered on the laser spot. The dotted line shows laser-On time for the temperature rise (black) profile.

Suppl. Fig. 3. Response of a cortical neuron exhibiting calcium spikes in response to the near infrared laser spot at-a-distance. (a) Representative time-lapse fluorescence images of a cortical neuron exhibiting calcium spikes in response to the laser spot (10 mW, 1000 nm) at-a-distance (indicated by red spot). Scale bar represents 15 µm. (b) Integrated fluorescence intensity (arbitrary units) versus time for soma (black profile) and upper right neurite (red profile) marked by white arrow in panel a.

Suppl. Fig. 4. Calibration of microheater and simulations of current-driven temperature gradient. (a) Image of a micro-heating device. (b) Electrical resistance of the titanium micro-heater line measured as a function of the ambient temperature. The fitted line provides the calibrated values of temperature from measured electrical resistance. (c) Temperature as a function of distance away from the micro-heater for

six different times. (d) Peak steady-state temperature in the micro-heater device as a function of electric current. For these simulations, the width of the titanium micro-heating element is set at 60 μm (matching the experimental condition) and surrounding medium as water. x = 0 corresponds to micro-heater-medium interface.

Suppl. Fig. 5. Illustration of proposed (photo)thermal mechanisms involved in (a) attractive and (b) repulsive axonal guidance. The red spot indicates laser spot. Red and orange squares represent activated temperature-sensitive and mechanically-sensitive ion channels respectively through which calcium ions are introduced to the growth cone. (c) Dependence of laser-induced axonal guidance parameters on distance of growth cone from the laser spot. Blue line represents value of direct forcing and/or stabilization of intracellular actin and/or filopodia. Orange line represents activity of mechanically sensitive (stretch-activated) calcium ion channels and the red line represents the temperature gradient due to absorption of laser light by medium and intracellular components (or by direct heating). The black line separates the attractive and repulsive regimes based on distance of growth cone from the laser spot.

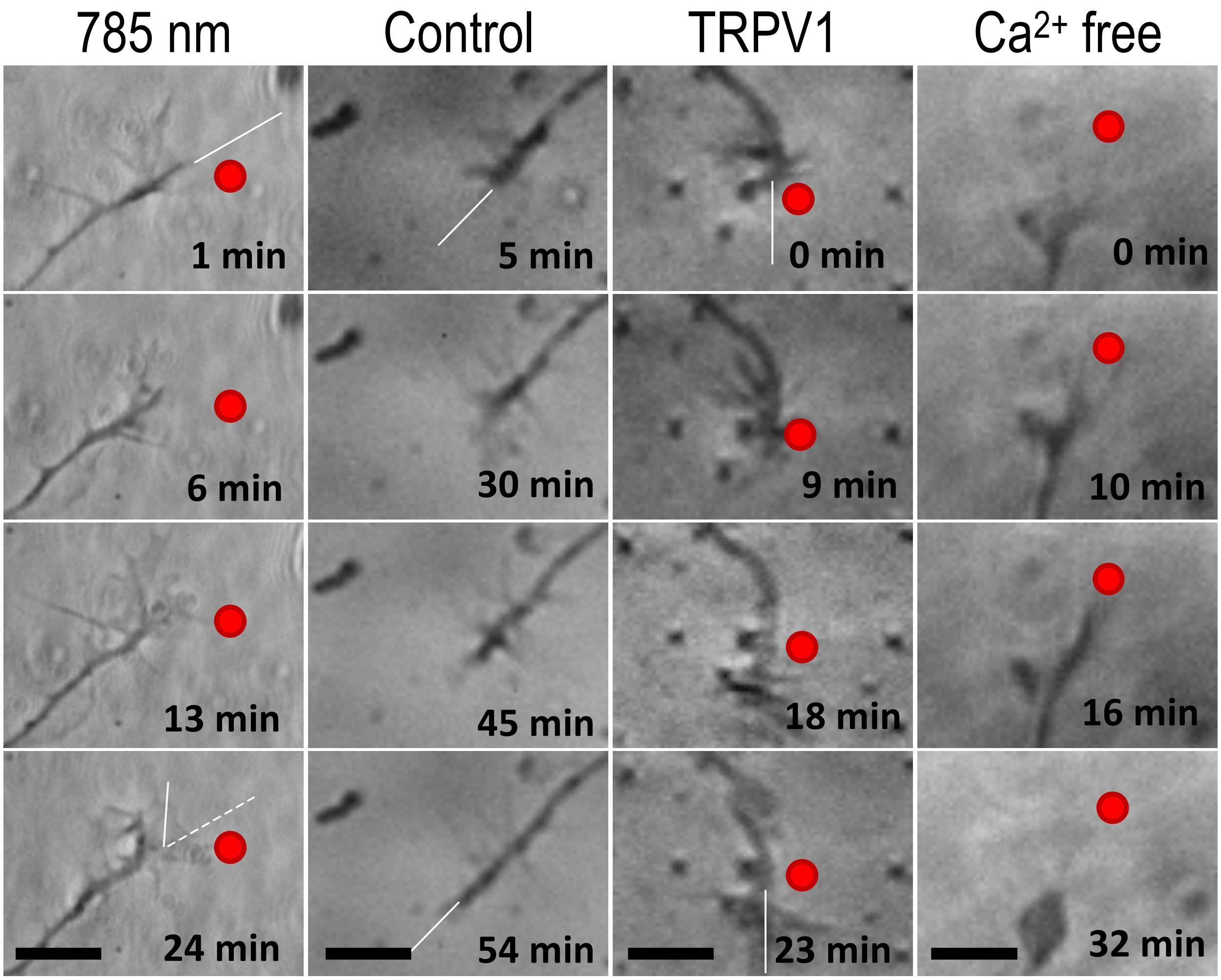

Suppl. Figure 1

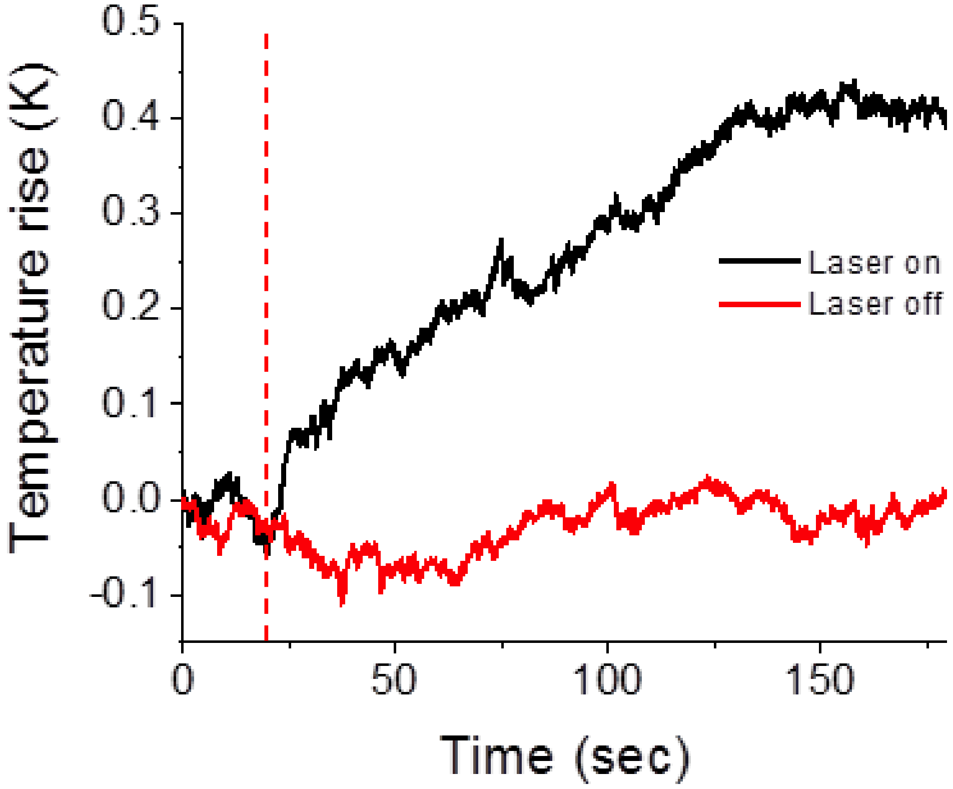

Suppl. Figure 2

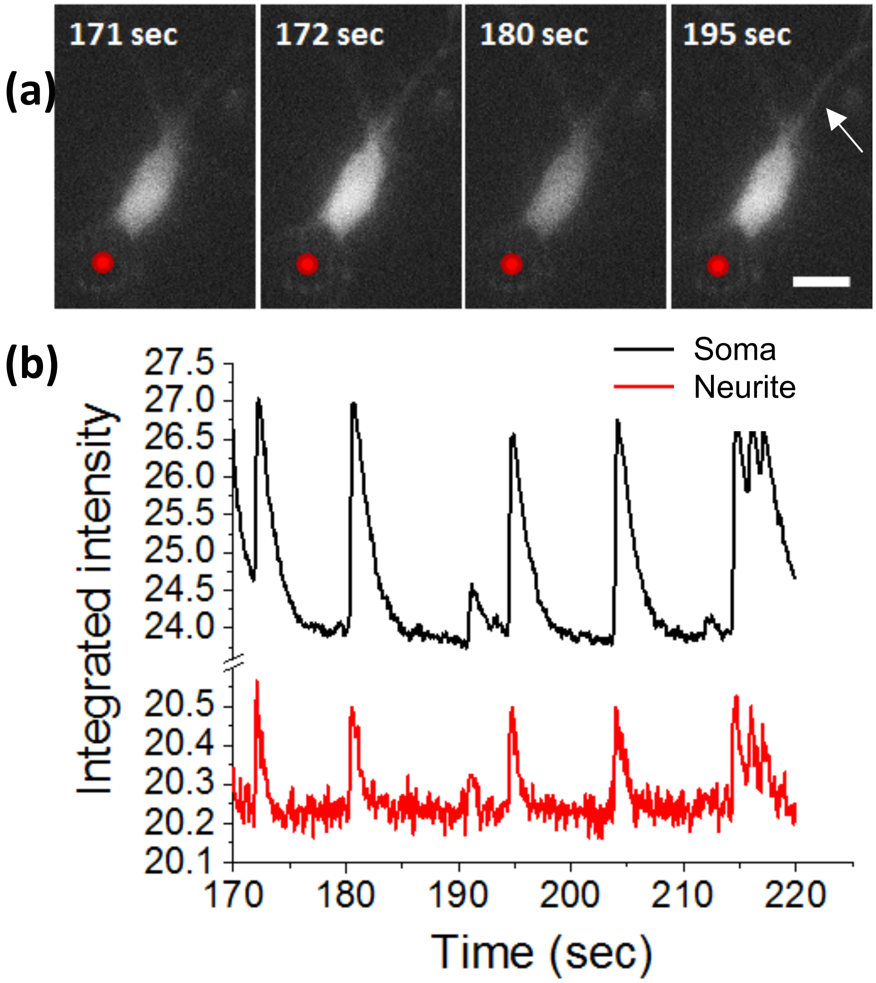

Suppl. Figure 3

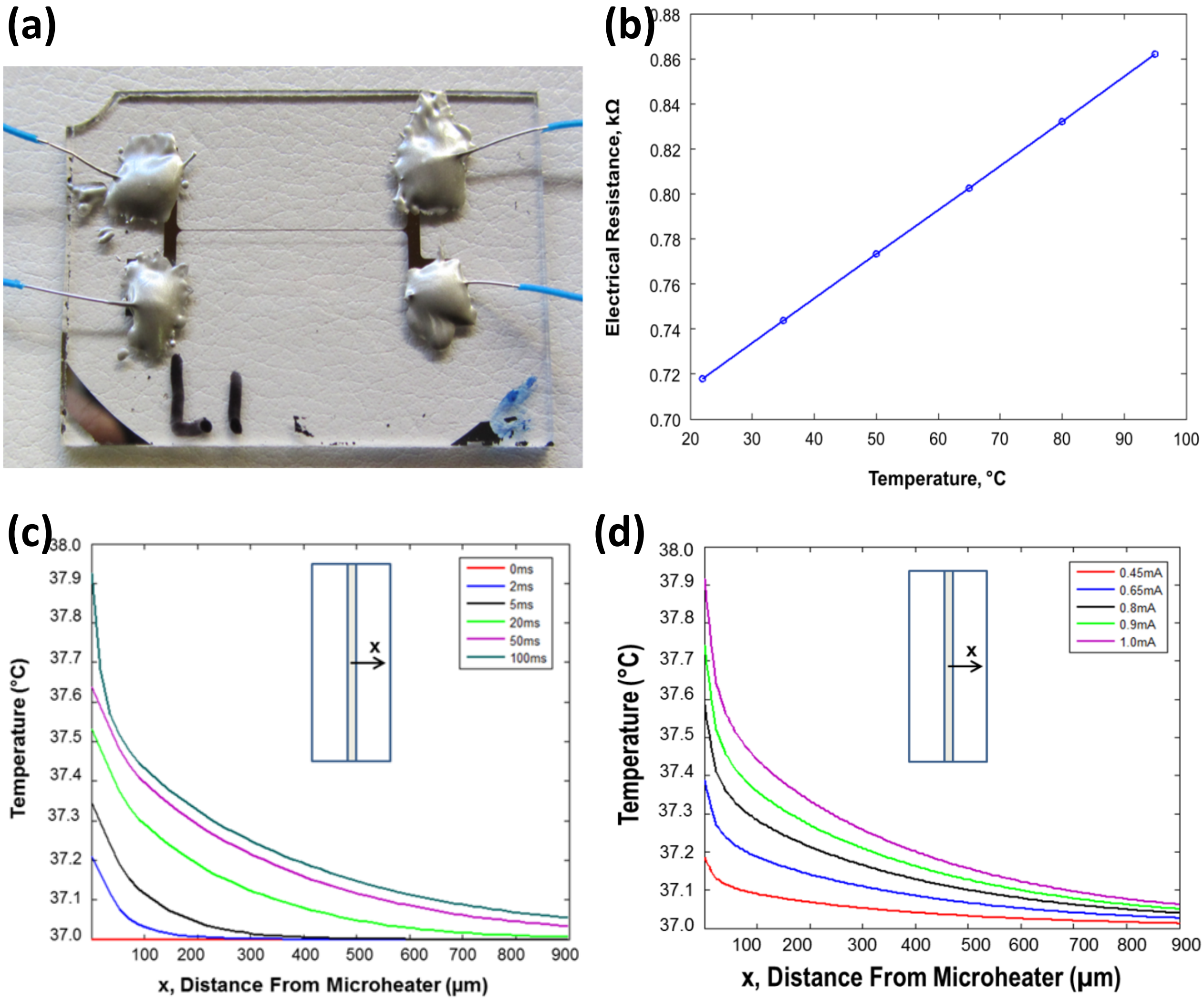

Suppl. Figure 4

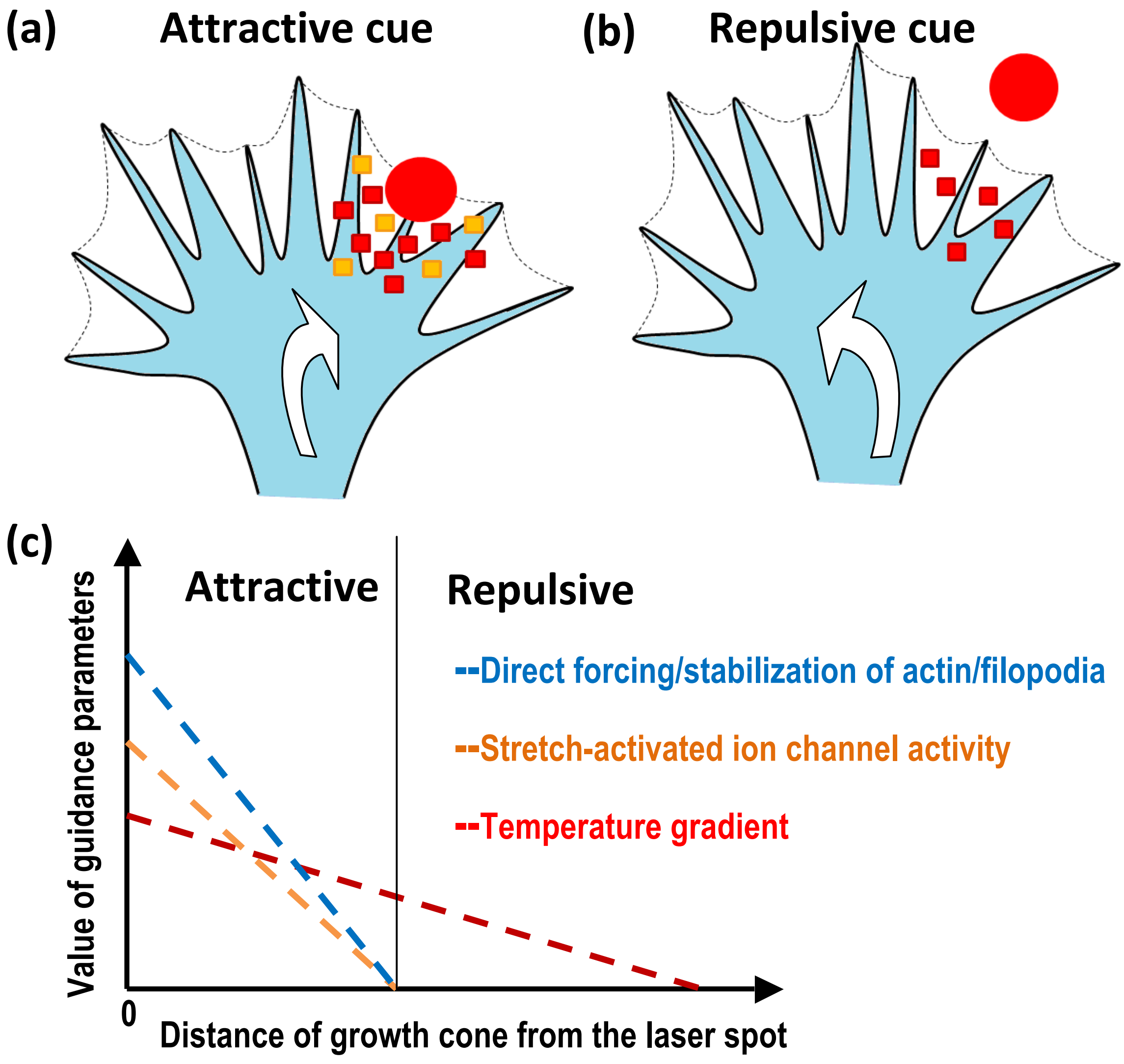

Suppl. Figure 5
